# Effects of Dietary *Macleaya cordata* Extract on Growth Performance, Biochemical Indices, and Intestinal Microbiota of Yellow-Feathered Broilers Subjected to Chronic Heat Stress

**DOI:** 10.3390/ani12172197

**Published:** 2022-08-26

**Authors:** Mingcan Wang, Junkai Zhang, Xiuqiong Huang, Yisong Liu, Jianguo Zeng

**Affiliations:** 1 College of Veterinary Medicine, Shanxi Agricultural University, Jinzhong 030801, China; 2 Hunan Key Laboratory of Traditional Chinese Veterinary Medicine, Hunan Agricultural University, Changsha 410128, China

**Keywords:** *Macleaya cordata* extract, broilers, growth performance, microbiota, biochemical parameter

## Abstract

**Simple Summary:**

Heat stress severely reduces poultry production performance and affects animal welfare. *Macleaya cordata* extract is a potential anti-stress product. To date, the effect of *Macleaya cordata* extract on heat stress in broilers is unclear. This research discussed the influence of *Macleaya cordata* extract supplementation on the performance, biochemical indices, and microbiota in heat-stressed yellow-feathered broilers. This will provide a scientific basis for the application of *Macleaya cordata* extract in animal husbandry under heat stress conditions.

**Abstract:**

This study investigated the effect of dietary *Macleaya cordata* extract (MCE) supplementation on the growth performance, serum parameters, and intestinal microbiota of yellow-feather broilers under heat stress. A total of 216 yellow-feather broilers (28-days-old) were randomly allotted into three groups. A control group (CON) (24 ± 2 °C) and heat stress group (HS) (35 ± 2 °C) received a basal diet, and heat-stressed plus MCE groups (HS-MCE) (35 ± 2 °C) were fed the basal diet with 1000 mg/kg MCE for 14 consecutive days. The results revealed that MCE supplementation improved the final body weight, average daily feed intake, average daily gain, and spleen index when compared with the HS group (*p* < 0.05). In addition, MCE supplementation decreased (*p* < 0.05) the activities of aspartate aminotransferase, alanine aminotransferase, lactate dehydrogenase, and creatinine, and increased (*p* < 0.05) the glucose level and alkaline phosphatase activity in heat-stressed yellow-feathered broilers. Moreover, MCE treatment alleviated heat-stress-induced intestinal flora disturbances, decreased the Bacteroidota and *Bacteroides* relative abundances, and increased Firmicutes. A linear discriminant analysis effect size analysis found five differentially abundant taxa in the HS-MCE group, including *Alistipes*, Rikenellaceae, *Mogibacterium*, *Butyrivibrio*, and *Lachnospira*. These results suggest that MCE can alleviate HS-induced decline in growth performance by modulating blood biochemical markers and cecal flora composition in broilers.

## 1. Introduction

Heat stress (HS) is one of the main stressors in poultry production. HS reduces broiler performance, affects food safety, and causes more than $128 million USD in economic losses each year [1,2,3]. HS reduces animal feed intake; affects the nutrient utilization, nutrient absorption, and barrier function in the gut; increases the proliferation of pathogenic gut microbiota; and causes intestinal damage and the translocation of pathogens and antigens into blood circulation, consequently seriously affecting animal health [4]. HS leads to oxidative stress, demonstrated by excessive reactive oxygen species (ROS) production and impaired antioxidant capacity [5]. Oxidative stress can induce damage to biological macromolecules such as DNA, proteins, lipids, etc., which in turn leads to cell dysfunction and tissue damage [6]. In addition, HS also leads to inflammation, and damages the intestinal barrier, resulting in dysbiosis [7]. The composition and function of the gut microbiota are critical to host health [8]. The gut microbiota can also influence the brain–gut axis to regulate host metabolic homeostasis, health, and behavior through microbiota-derived metabolites, hormones, and neurotransmitters [9]. Therefore, the regulation of gut microbiota homeostasis by inhibiting oxidative stress and/or inflammation in organisms is important for preventing and/or treating HS.

Yellow-feather broilers are a broiler breed with a long history in China. They have a slow growth rate, are succulent and refined, and have good chicken flavor quality, so they are widely welcomed by consumers in China and Southeast Asia. The feeding cycle of yellow-feathered broilers is 130–160 days, the weight of adult chickens can reach 1.6–1.8 kg, and the feed conversion ratio (FCR) of the whole feeding period is about 3.2 g/g. Like other common breeds of broilers, HS seriously affects the health status of yellow-feathered broilers, including the growth performance, immune function, carcass traits, meat quality, flora homeostasis, etc. [10,11,12].

Studies have shown that many plants’ bioactive compounds, including traditional Chinese medicine, improve intestinal aerobiosis and ameliorate mucosal barrier dysfunction, especially during stress conditions [4]. *Macleaya cordata* (family *Papaveraceae*) is a perennial herb and traditional Chinese medicine that is widely distributed in the south of China. *Macleaya cordata* extract (MCE) is an orange-yellow powder made from the fruit pods of *Macleaya cordata* (Willd.) R. Br., which is a natural, pollution-free green product. The MCE standardized extraction process is referenced by Dong (2022) [13]. In a previous study, five species of MCE components were identified, including Benzophenanthridine, Benzyltetrahydroisoquinoline, Protopine, Protoberberine, and Tetrahydroptotoberberine, and the specific components refer to Dong (2021) [14]. The main components of MCE are sanguinarine and chelerythrine. Both sanguinarine and chelerythrine belong to Benzophenanthridine. It has been reported that sanguinarine and chelerythrine have physiological effects, such as antibacterial, anti-inflammatory, and anti-tumor effects, as well as enhancing immunity [15,16,17,18]. Sanguinarine and chelerythrine are considered excellent animal feed additives due to their unique pharmacological properties and health benefits [19,20]. Previous studies indicated that dietary supplementation with MCE could improve the growth performance of grass carp [21], weaned pigs [22], cattle [23], and broilers [24]. The dietary supplement, MCE, has previously been reported to improve growth performance, antimicrobial activity, intestinal luminal environment, and gut development in weaned piglets [25,26].

However, whether MCE can alleviate the negative effects of HS on poultry is unknown. The purpose of this study was to explore the effects of MCE on HS-induced growth performance, blood biochemical markers, and gut microbiota in broilers.

## 2. Materials and Methods

### 2.1. Experimental Design and Diets

The experimental design was referred to in He et al. (2018) [27]. A total of 216 28-day-old male yellow-feathered broiler chicks were randomly and equally assigned to three groups, with 72 chickens per group. There were 6 replicates per group, with 12 chicks per replicate that were housed in cages measuring 180 cm × 90 cm. The birds in the control group (CON) and heat stress group (HS) were fed a basal diet, while the *Macleaya cordata* extract group (HS-MCE) was fed a basal diet supplemented with a combination of 1000 mg/kg of MCE. MCE contains more than 0.375% sanguinarine and chelerythrine, and other components of starch. MCE was purchased from Hunan Meikeda Biological Resources (Changsha, China). The MCE dose conversion is referenced in a previously published study [28]. Based on recommendations from the Ministry of Agriculture of the People’s Republic of China (2004), the basal diets (Table 1) were formulated. Among them, the CON group was housed in an environmentally-controlled chamber at 24 ± 2 °C, and the HS and HS-MCE groups were housed in an environmentally-controlled chamber at 35 ± 2 °C (from 09:30 to 17:30), and the rest of the day the temperature was controlled at 24 ± 2 °C. A 28-day-old broiler was tested for 14 consecutive days, receiving ad libitum feed and water during the trial. This feeding experiment was conducted in the laboratory of the Institute of Animal Science, Guangdong Academy of Agricultural Sciences.

### 2.2. Growth Performance and Organ Indices

In each replicate, the initial body weight, final body weight, and feed intake of each group were recorded, and the average daily gain (ADG), average daily feed intake (ADFI), and feed conversion ratio (FCR) were calculated.

At the end of the experiment, a chicken with a weight close to the average weight of each replicate was selected, and the serum and cecum contents were collected and stored in a −80 °C refrigerator for later use; additionally, the liver, bursa, and spleen were separated, and the organ index was calculated.

### 2.3. Analyses of Serum Biochemical Indicators

The serum was obtained by centrifuging all of the whole blood at 3000× *g* for 15 min at 4 °C. The serum biochemical indicators in Table 1 were analyzed using a Biochemical Analyzer ZY400 (KEHUA, Shanghai, China). These serum biochemical indices were measured with corresponding reagent kits that were all from KEHUA Biotechnology Co., Ltd. (Shanghai, China).

### 2.4. Microbial Analysis

Using the SDS method, DNA from the cecal contents were extracted. The purity and concentration of the DNA were determined using agarose gel electrophoresis. The sample amplification methods were referred to in Liu (2020) [7]. MiSeq metagenomic sequencing was completed at Wuhan MetWare Biomedical Technology Co., Ltd. (Wuhan, China), and the bacterial diversity index analysis was performed on its cloud platform.

### 2.5. Statistical Analysis

The data were analyzed using SPSS 24.0 software (IBM Corp, Armonk, NY, USA). A one-way analysis of variance (ANOVA) followed by Tukey’s multiple comparison test was used to determine the significance of the data. The means and standard errors are used to express data. The statistical significance of differences was expressed as *p* < 0.05 or *p* < 0.01.

## 3. Results

### 3.1. Effects of HS and MCE on Growth Performance

Table 2 shows the effect of MCE on heat-stress-induced broiler growth performance. HS significantly reduced Final BW, ADFI, and ADG in broilers compared to the CON group (*p* < 0.05). The addition of MCE to the diet resulted in a significantly higher Final BW, ADFI, and ADG in broilers than in the HS group (*p* < 0.05), yet it was significantly lower than the CON group. However, there were no significant differences in feed conversion ratio (FCR) among the three groups (*p* > 0.05).

### 3.2. Effects of HS and MCE on Organ Indices

As shown in Table 3, there were no significant differences in the Bursa of Fabricius index among the three groups (*p* > 0.05). The Spleen index and the Liver index in the HS group were significantly lower than in the CON group (*p* < 0.05). In the HS-MCE group, the Spleen index was significantly higher (*p* < 0.05) and the Liver index tended to increase compared with the HS group (*p* = 0.07).

### 3.3. Effects of HS and MCE on Serum Biochemical Indicators

The results of the serum biochemical indicators of yellow-feathered broilers are shown in Table 4. Compared with the CON group, the HS treatment increased (*p* < 0.05) the serum aspartate aminotransferase (AST), alanine aminotransferase (ALT), and lactate dehydrogenase (LDH) activity; increased the cholesterol (TC) and low-density lipoprotein (LDL-C) concentrations; and decreased (*p* < 0.05) the serum alkaline phosphatase (ALP) and glucose (GLU) concentrations; however, the creatinine and total protein (TP) concentrations did not change significantly (*p* > 0.05). In the HS-MCE group, the serum AST, ALT, and LDH activity, and creatinine concentrations were significantly decreased (*p* < 0.05), and the ALP and GLU concentrations were significantly increased compared with the HS group (*p* < 0.05). No significant differences in the uric acid, TP, and high-density lipoprotein (HDL-C) concentrations were seen among the three treatments.

### 3.4. Effects of HS and MCE on Intestinal Microbiota

**Figure 1:**
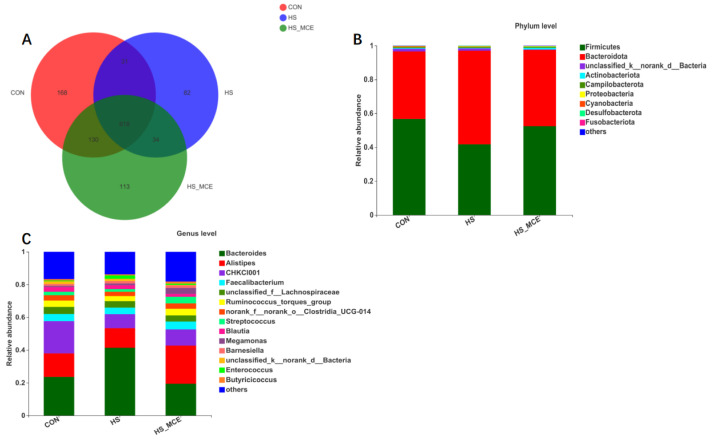
The Venn diagram and bacterial community composition of yellow-feathered broilers. (**A**) The Venn diagram of OTUs. (**B**) Microbial composition in the cecum at the phylum level. (**C**) Microbial composition in the cecum at the genus level. Abbreviations: CON, control group; HS, heat stress group; HS-MCE, Heat Stress plus MCE group.

In Figure 1, based on a 97% similarity, the sample sequence was clustered according to OTUs. After identification, there were a total of 1176 OTUs in the sample. Among them, the OTU numbers of the CON, HS, and HS-MCE groups were 947, 765, and 895, respectively (Figure 1A). At the phylum level (Figure 1B), the dominant phyla are Firmicutes and Bacteroidetes. HS increased the relative abundance of Bacteroidetes and decreased the relative abundance of Firmicutes in broilers. Supplementation with MCE resulted in a return of the relative abundances of Bacteroidetes and Firmicutes to their original levels. The dominant microbiota in the cecal contents at the genus level (Figure 1C) consisted of *Bacteroides* and *Alistipes*. HS significantly increased the *Bacteroides* and decreased the *Alistipes* compared to the CON group, whereas dietary MCE significantly decreased *Bacteroides* and increased *Alistipes* compared to the HS group.

**Figure 2:**
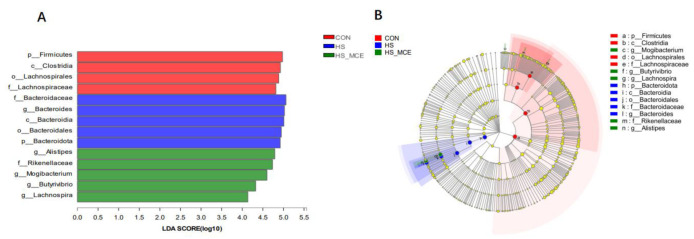
Linear discriminant analysis (LDA) effect size (LEfSe) analysis (*p* < 0.05, LDA > 4.0) of the intestinal microbes. (**A**) LDA. (**B**) LEfSe. The prefixes “p”, “c”, “o”, “f”, and “g” represent the annotated level of phylum, class, order, family, and genus. Abbreviations: CON, control group; HS, heat stress group; HS-MCE, Heat Stress plus MCE group.

In Figure 2, The LEfSe analysis yielded 14 biomarkers based on the LDA > 4. The CON group markers include Firmicutes (phylum), Clostridia (class), Lachnospirales (order), and Lachnospiraceae (family). Moreover, Bacteroidaceae (family), *Bacteroides* (genus), Bacteroidia (class), Bacteroidales (order), and Bacteroidota (phylum) were enriched in the HS group, while *Alistipes* (genus), Rikenellaceae (family), *Mogibacterium* (genus), *Butyrivibrio* (genus), and *Lachnospira* (genus) were enriched in the HS-MCE group.

**Figure 3:**
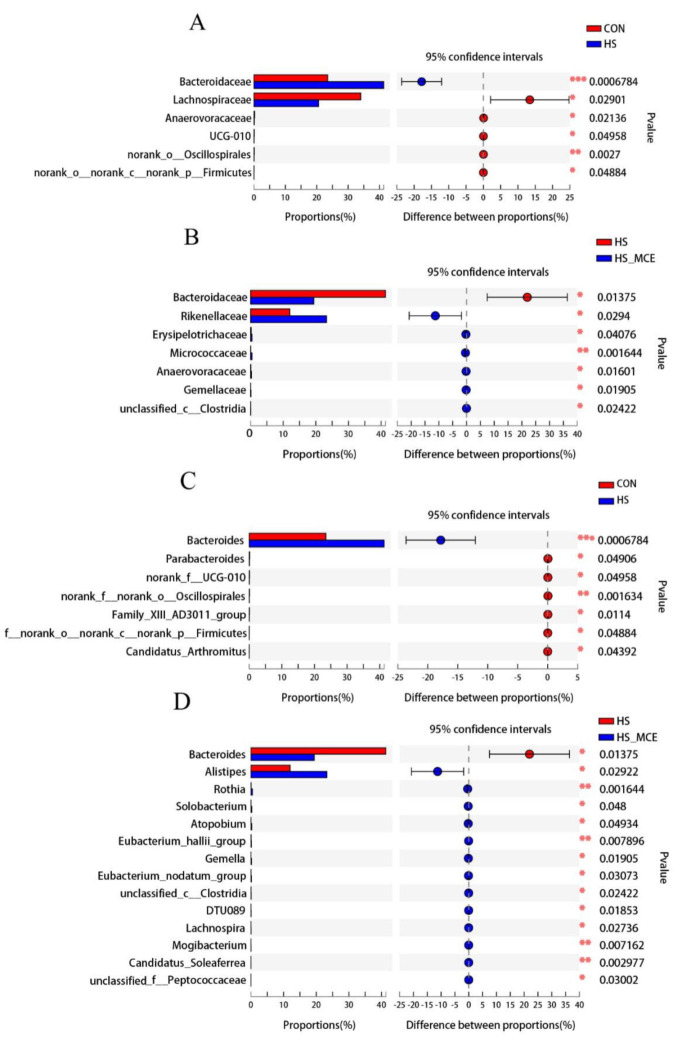
T−test analysis for the significant changes of differential microbiota at the family and genus level in the cecal contents of yellow-feathered broilers. (**A**) CON vs. HS at family level. (**B**) HS vs. HS-MCE at family level. (**C**) CON vs. HS at genus level. (**D**) HS vs. HS-MCE at genus level. Abbreviations: CON, control group; HS, heat stress group; HS-MCE, Heat Stress plus MCE group. * indicates statistically significant difference (*p* < 0.05). ** indicates extremely significant difference (*p* < 0.01). *** indicates very extremely significant difference (*p* < 0.001).

Figure 3 shows the compositional differences at the family and genus levels between groups. HS increased the relative abundance of Bacteriaceae (family) and decreased the abundance of Lachnospira (family), while the relative abundance of *Bacteroides* (genus) was increased compared with the CON group. Moreover, in the HS-MCE group, a lower relative abundance of Bacteroidaceae (family) and *Bacteroides* (genus) and higher abundance of Rikenellaceae (family) and *Alistipes* (genus) was found compared to the HS group.

**Figure 4:**
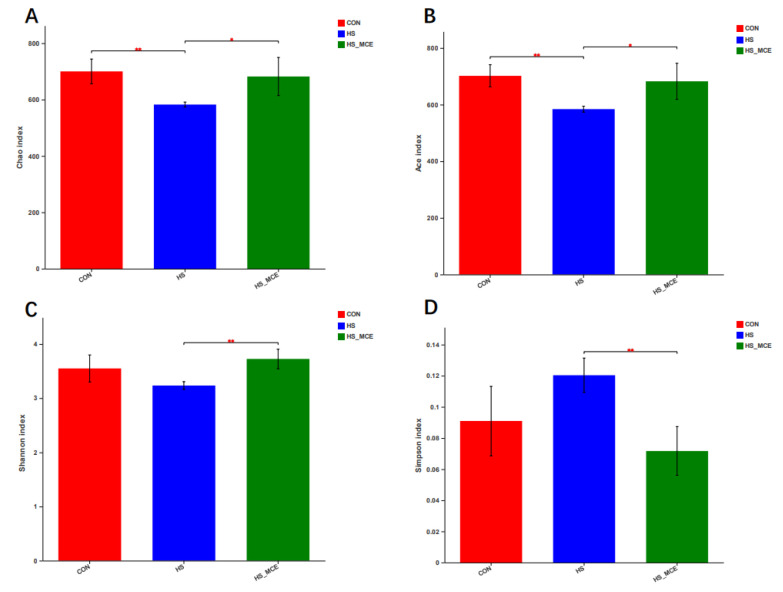
Cecal bacterial Alpha diversity of yellow-feathered broilers. (**A**) Chao index. (**B**) Ace index. (**C**) Shannon index. (**D**) Simpson index. Abbreviations: CON, control group; HS, heat stress group; HS-MCE, Heat Stress plus MCE group. * indicates statistically significant difference (*p* < 0.05). ** indicates extremely significant difference (*p* < 0.01).

Figure 4 shows the effect of MCE supplementation on the microbiota diversity of heat-stressed broilers. HS significantly reduced the Chao and Ace indices compared with the CON group (*p* < 0.01). In the Shannon and Simpson indices no significant differences were found between the CON and HS groups (*p* > 0.05). However, MCE supplementation significantly increased the Chao (*p* < 0.05), Ace (*p* < 0.05), and Shannon (*p* < 0.01) index compared with the HS group, where the trend in the Simpson index is the opposite.

**Figure 5:**
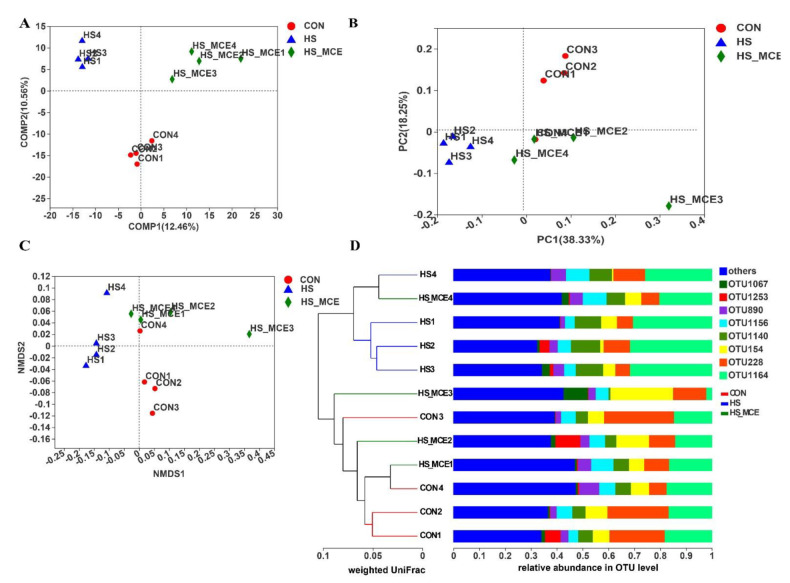
Cecal bacterial Beta diversity of yellow-feathered broilers. (**A**) PLS−DA analysis. (**B**) PCoA analysis. (**C**) NMDS analysis. (**D**) Hierarchical cluster analysis. Abbreviations: CON, control group; HS, heat stress group; HS-MCE, Heat Stress plus MCE group.

Figure 5 shows the microbial beta diversity. According to PLS-DA based on Bray-Curtis distance, there was a clear clustering among the CON, HS, and HS-MCE groups. Moreover, we obtained a similar result in PCoA and NMDS (Figure 5B,C). To further investigate the similarity of bacterial genes, a hierarchical cluster was used based on the UniFrac distance matrix and confirmed the distinct microbial communities among the CON, HS, and HS-MCE groups. As shown in Figure 5D, CON group samples CON1, 2, 3, and 4 and HS-MCE group samples HS-MCE1, 2, and 3 are clustered into one category, and HS group samples HS1, 2, and 3 and HS-MCE4 are clustered into one category. After treatment with MCE, HS-MCE1, 2, and 3 can be well clustered with CON1, 2, 3, and 4. However, the HS-MCE4 and HS group samples are clustered together.

## 4. Discussion

HS has caused huge economic losses to the livestock industry [29]. Research shows that HS severely affects animal health and performance [30]. In our current research, the Final BW, ADFI, and ADG in the HS group were significantly lower than in the CON group, suggesting the harmful effects of HS on chickens. This is consistent with previous findings that acute HS resulted in reduced body weight and feed intake when birds were exposed to 31 °C for 10 h [31]. The increased respiration rate may reduce the bird’s feed intake due to the absence of sweat glands [32]. In addition, anorexia hormone secretion under HS may also lead to reduced feed intake [33]. The reduction in body weight can be attributed to a reduction in feed intake. Supplementation with MCE has been shown to improve performance and immune function in weaning pigs, Xuefeng black-bone chicken, and Pacific White Shrimp [25,34,35]. However, the effects of MCE on animal performance were not identical in different experiments. It has been reported that dietary MCE administration increased the Final BW and ADG, and the reduced feed efficiency and diarrhea rate in weaned piglets [25]. Moreover, diets with 0.15 g/kg of MCE increased the feed intake and weight gain on day 24 and day 35, and improved FCR, flock uniformity, and breast meat yield on day 35 in broilers with necrotizing enteritis [17]. In contrast, MCE supplementation did not alter production performance and inflammation levels, but rather reduced MDA contents, increased SOD activities in both the ileum and jejunum, increased GSH-Px activity in the jejunum, and attenuated oxidative damage in weaned goats [36]. In the present study, we found that dietary supplementation with MCE increased the Final BW, ADFI, and ADG in heat-stressed broilers; however, there were no significant differences in FCR among the three groups. The small intestine is the main site of animal digestion and absorption, and HS causes damage to the small intestinal mucosa [37]. The sanguinarine [38] and chelerythrine [39,40] in MCE can reduce intestinal inflammation and repair intestinal mucosal damage, which may partially explain the effect of MCE on reducing HS-induced performance damage in yellow-feathered broilers. The unaltered FCR may be due to the environment, among other reasons, thereby resulting in a lower ratio of the increased ADFI to meat product than the normal FCR. Variations between studies could be due to many factors, including different additions, breed, age, and different experimental conditions. Notably, although supplementation with MCE improved Final BW, ADFI, and ADG in yellow-feathered broilers compared to the HS group, the performance was still not as good as the CON group. This may be due to the fact that HS acts on broilers for too long, resulting in the disorder of the body’s hormone levels and serious damage [41]. The dose of MCE in this experiment didn’t completely offset the negative effects of HS on broilers. Similar studies showed that MCE also did not fully restore the small intestine bacterial burden and serum antioxidant enzyme activity of mice challenged with enterotoxigenic *Escherichia coli* [42]. The optimal dose of MCE for preventing HS-induced injury in broilers needs further exploration.

The liver is an important metabolic organ of the body, and the Bursa of Fabricius and spleen are the immune organs of poultry. Studies have shown that HS induces inflammation and oxidative stress leading to a decreased Spleen index, Bursa index [43], and liver weight [44]. In this research, HS decreased the index of the spleen and liver, while the Bursa of Fabricius index did not change compared with the CON group. Moreover, previous research reported that supplementation of sanguinarine in the diet of laying hens can improve cellular and humoral immunity [45]. In our current research, dietary supplementation with MCE significantly increased the Spleen index in broilers during HS, indicating that MCE reduced the body’s inflammatory response and oxidative stress damage and enhanced immune function in HS-induced broilers. The specific reason for the increased Liver and Spleen index caused by MCE may be that sanguinarine/chelerythrine attenuates HS-induced inflammation and apoptosis of liver and spleen tissue or cells by inhibiting the TLR4/NF-κB signaling pathway or inhibiting exudate and prostaglandin E2 production and release by regulating cyclooxygenase-2 [39,46].

Biochemical indices in blood reflect the health and metabolic status of animals. Creatinine and uric acid are nitrogenous and proteinaceous end products of catabolic processes excreted by the kidneys and are reliable indicators of renal function [47]. When the kidneys are damaged, the levels of creatinine and uric acid in the blood rise significantly [48]. There are research reports that HS induces renal damage in poultry [49]. Consistent with previous studies [50], this experiment found that HS led to a significant increase in serum creatinine levels compared with the CON group, which suggested that HS could damage the kidney function of yellow-feathered broilers. In this regard, sanguinarine has previously been reported to reduce kidney damage in broilers at 28 days [24]. This is similar to our results. Our results showed that MCE decreased the creatinine in the HS-MCE group compared with the HS group, indicating that MCE may have a potent protective effect on renal dysfunction induced by HS. The decreased creatinine was consistent with the result that sanguinarine supplementation increased the levels of Gly, Ile, Lys, Met, Arg, Ala, and Thr in the serum of growing pigs [51]. These trends are consistent with growth performance, suggesting that sanguinarine is beneficial in increasing protein synthesis, resulting in a reduction in serum creatinine, uric acid, and urea nitrogen; an increase in body energy storage; and, ultimately, an increase in ADG. In addition, sanguinarine can be induced to irreversibly bind L-amino acid decarboxylase due to their structural similarity to aromatic amino acids such as tryptophan, phenylalanine, and tyrosine [52], thereby inhibiting intestinal aromatic amino acid decarboxylase [52,53,54] and improving serotonin synthesis in the liver, gut, and brain tissue [53]. In this way, sanguinarine increases the availability of aromatic amino acids [54], resulting in a reduction in the concentration of toxic biogenic amines [52]. In addition, studies have shown that another major component of MCE, chelerythrine, is a selective protein kinase C inhibitor [55]. Protein kinase C can lead to mitochondrial dysfunction and ischemic kidney injury [56]. Therefore, another reason for the decrease in serum creatinine levels may be due to the inhibition of protein kinase C by chelerythrine, which alleviates renal damage. It was reported that elevated serum alkaline phosphatase (ALP), lactate dehydrogenase (LDH), alanine transaminase (ALT), and aspartate transaminase (AST) are important indicators of liver function, and liver damage can lead to the abnormal activity of these enzymes [57]. A previous study showed that serum ALT and AST activities were increased under HS [58,59], and MCE (100 mg/L) can significantly inhibit the activities of ALT and AST in snail Oncomelania hupensis [60]. A report on the anti-fibrotic effects of Corydalis saxicola Bunting (CS) using a combined metabolomic and network pharmacology approach suggests that chelerythrine and sanguinarine are potentially active compounds in CS for the treatment of liver fibrosis by modulating ALT activity [61]. Consistently, the current study showed that treatment with MCE reduced the AST, ALT, and LDH activity and increased the ALP activity in HS-induced broilers, suggesting that MCE seems to protect the broilers from liver injury. The protective effect of MCE on the liver may be due to the inhibition of hepatic NAD(P)H quinone oxidoreductase activity [62] and the hepatic lipid peroxidation and inflammatory response [63] by chelerythrine. Moreover, HS also significantly decreased the levels of serum GLU and significantly increased the levels of TC and LDL-C. Similarly, research has shown that HS alters carbohydrate and fat metabolism, increases serum TC and LDL-C levels, and decreases GLU levels [64,65]. The disturbance of glucose metabolism and fat metabolism caused by HS can be attributed to the activation of the hypothalamic-pituitary-adrenal (HPA) axis, which leads to an increase in plasma glucocorticoid concentration and ultimately leads to the disturbance of energy metabolism in the body [66,67]. However, studies have also reported that plasma GLU was significantly elevated during acute HS, and plasma GLU was not significantly different from controls during chronic HS [68]. These differences may be due to the animal’s breed, age, and duration of HS. Moreover, recent studies revealed that treatment with 200mg/kg of MCE effectively improved the GLU content but did not change the total cholesterol and triglyceride concentrations in Xuefeng black-bone chicken [34]. In our study, dietary MCE supplementation had significantly increased blood GLU content but did not significantly change TP, TC, LDL-C, and HDL-C concentrations compared with the HS group, indicating that the addition of MCE may promote the digestion and absorption of carbohydrates, but it has a minimal effect on protein and lipid metabolism in the diet. The recovery of GLU levels is consistent with the improvement in Final BW that we observed in the performance data. It has been reported that sanguinarine and chelerythrine can induce the accumulation of glucocorticoid receptors (GR) in the nucleus with a concomitant reduction of cytosolic GR, and inhibit the binding of corticosterone to GR, thereby alleviating the effect of glucocorticoids on carbohydrate metabolism [69], which may explain the recovery of blood glucose levels in broiler chickens in the HS-MCE group. The mechanism of the effect of MCE on lipid metabolism and protein metabolism needs further study.

It is generally accepted that a stable gut microbiome is beneficial to the body’s health [70]. At the phylum level, the intestinal flora of broilers is mainly Firmicutes and Bacteroidetes [71]. The study has shown that HS significantly alters gut microbiota composition and structure in broilers and layers [72,73,74]. In this research, the number of OTUs (765) in the HS group was significantly lower than that of those in the CON group (947), and, after MCE treatment, the number of OTUs (895) in the HS-MCE group recovered to close to the level of the CON group. Furthermore, HS resulted in a significant increase in the relative abundance of Bacteroidota (phylum) and *Bacteroides* (genus), while there was a significant decrease in the relative abundance of Firmicutes (phylum) in broilers, and MCE significantly alleviated this trend compared with the HS group. This is consistent with previous findings that HS led to an increase in Bacteroidetes and a decrease in Firmicutes in the gut of laying hens [74], whereas sanguinarine, the main component of MCE, significantly reduced Bacteroidetes and increased Firmicutes in broilers [24]. This suggests that MCE can alleviate the disturbance of gut flora structure caused by HS in yellow-feathered broilers. In this experiment, the HS group used the Bacteroidota (phylum), Bacteroidales (order), Bacteroidia (class), *Bacteroides* (genus), and Bacteroidaceae (family) as markers; and the HS-MCE group used the *Alistipes* (genus), Rikenellaceae (family), *Mogibacterium* (genus), *Butyrivibrio* (genus), and *Lachnospira* (genus) as markers. This result can be further confirmed by Welch’s t-test. *Bacteroides* belong to anaerobic gram-negative bacilli that produce SCFAs that are beneficial to health [75], and the abundance of *Bacteroides* is negatively correlated with FCR [76]. In this study, the reason for the increase in *Bacteroides* caused by HS is not clear, though increased *Bacteroides* may be a form of body self-regulation of HS. However, the role of *Bacteroides* in different environments needs to be studied further. *Alistipes* are beneficial to the growth of broilers [77]. Rikenellaceae are associated with a healthy metabolism in the body [78]. An increase in *Mogibacteriaceae* is associated with a significant reduction in cecal inflammation in mice [79]. *Butyrivibrio* is linked to recovery from necrotizing colitis in broilers [80]. *Lachnospira* can reportedly degrade starch and non-starch polysaccharides to produce organic acids [81], thereby decreasing the expression of virulence factors from pathogens like Salmonella [82]. HS is known to cause inflammation, an increase in harmful intestinal bacteria, and growth inhibition in poultry [83,84]. Interestingly, these dominant probiotics in the HS-MCE group had functions such as enhancing digestion and absorption, promoting growth, reducing inflammation, and inhibiting the growth of harmful bacteria. MCE may alleviate HS in broilers by increasing the abundance of these beneficial bacteria, thereby indicating that the increased Final BW, ADFI, and ADG may be due to the increased probiotics in the HS-MCE group. In fact, there are indeed studies showing that MCE can promote animal growth by regulating intestinal flora [24]. HS-elevated corticosterone levels in the body can affect gut health and lead to dysbiosis of microbiota homeostasis [66,85]. Sanguine and chelerythrine can induce the accumulation of GR in the nucleus while reducing the cytoplasmic GR and inhibiting the binding of corticosterone to GR [69], which may ultimately alleviate the structural disturbance of the gut microbiota caused by HS.

The Chao, Ace, Simpson, and Shannon indices reflect the richness and diversity of the intestinal microbiota. In previous research, HS significantly increased ileal microbial alpha diversity, which was expressed as a higher number of observed species, Chao, and whole-tree phylogenetic diversity [84]. In addition, HS also increased growing pig fecal species richness [86]. However, HS has also been reported to reduce broiler cecal and tracheal microbiome alpha diversity, which has been expressed as a lower number of observed species and Chao and Shannon indices [87]. In two other studies, HS didn’t alter the alpha diversity of the gut microbiota in laying and broiler chickens [7,88]. This study found that HS led to a significant decrease in alpha diversity (Chao and Ace indexes). However, dietary MCE supplementation increased the alpha diversity (Chao, Ace, and Shannon indices) in the HS-MCE group. The Simpson index was contrary to the trend of other indices. This suggests that MCE can restore the disturbance of the diversity and richness of intestinal flora in broilers caused by HS. The conflicting results of HS on the intestinal microbiota alpha diversity might be associated with several factors, including the species, duration, intensity of exposure, and gut segments. PLS-DA was used to analyze the bacterial community structure. The more similar the sample composition, the closer the space in the PLS-DA plot. In our current study, every group’s samples are clustered into one category, respectively, and the distances between the three groups are quite different in the PLS-DA plot. PLS-DA revealed that the microbial composition and structure among the CON, HS, and HS-MCE groups are different, and HS may destroy the bacterial structure. We obtained the same result in subsequent PCoA and NMDS analyses. In addition, using hierarchical clustering based on the UniFrac distance matrix showed that the CON and HS-MCE groups had more similar bacterial genes, while the HS group had differences in the bacterial genes from the other groups. This suggests that HS alters the composition and structure of gut microbes in broilers, and MCE can alleviate HS-induced flora disturbances to a certain extent. These results are consistent with previous studies that suggest that HS significantly altered the bacterial community structure in the intestinal mucosa of broilers [89], while sanguinarine can regulate the structure of intestinal flora in broilers [24]. The inhibitory effects of different concentrations of MCE on the same flora were significantly different, and the effects of the same concentration of MCE on the flora of different species were also different. Studies have shown that MCE has the strongest inhibitory effect on Bacillus subtilis and Bacillus licheniformis, and the weakest inhibitory effect on *Lactobacillus* [90]. In this study, the restoration effect of MCE on HS-induced broiler gut microbiota structure may be due to the differences in tolerance of different microbiota to MCE. Probiotics such as *Alistipes*, Rikenellaceae, *Mogibacterium*, *Butyrivibrio*, and *Lachnospira* may have a higher tolerance to MCE, but MCE decreased the other harmful bacteria or opportunistic pathogens and indirectly led to the increase of probiotics due to the occupancy effect of the flora, which altered the HS-induced gut microbiota structure disturbances. The specific mechanism by which MCE alters the structure of the gut microbiota requires further study.

## 5. Conclusions

In conclusion, the study indicates that the growth performance and serum metabolites of broilers were negatively affected by HS. Furthermore, HS leads to decreased gut microbiota diversity and structural disturbances. Dietary MCE supplementation could have beneficial effects on the growth, metabolism, and flora homeostasis of yellow-feathered broilers under HS.

## Figures and Tables

**Table 1:** The ingredient and nutrient level of the basal diet.

Item	Amount
Ingredient (%)	
Corn	63.26
Soybean meal	28.00
Corn gluten meal	2.00
Soybean oil	2.50
Limestone	1.41
Dicalcium phosphate	1.33
DL-Met	0.15
L-Lys-HCl	0.18
Wheat middling	0.17
Vitamin-mineral premix ^1^	1.00
Calculated nutrient composition ^2^	
ME (MJ/kg)	12.54
CP (%)	18.63
Lys (%)	1.00
Met (%)	0.46
Ca (%)	0.88
Available *p* (%)	0.40

Notes: ^1^ The premix provided the following per kg of diet: VA, 6000 IU; VD3, 2000 IU; VE, 30 mg; VK3, 2 mg; VB1, 3 mg; VB2, 5 mg; pantothenic acid, 800 mg; choline chloride 1500 mg; nicotinic acid, 30 mg; pyridoxine, 3 mg; folic acid, 500 mg; biotin, 0.2 mg; VB12, 1 mg; Fe, 100 mg; Cu, 8 mg; Mn, 100 mg; Zn, 100 mg; I, 0.42 mg; Se, 0.3 mg. ^2^ Values were calculated from data provided by Feed Database in China (2004).

**Table 2:** Effects of HS and MCE on growth performance of yellow-feathered broilers.

Item	Treatment		
	CON	HS	HS-MCE
Initial BW, g	365.00 ± 0.29	365.06 ± 0.32	365.33 ± 0.40
Final BW, g	838.88 ± 6.29 ^a^	745.88 ± 11.81 ^c^	769.38 ± 2.47 ^b^
ADFI, g/d	64.21 ± 1.22 ^a^	51.61 ± 0.99 ^c^	54.86 ± 0.59 ^b^
ADG, g/d	33.85 ± 0.43 ^a^	27.20 ± 0.84 ^c^	28.85 ± 0.17 ^b^
FCR, g/g	1.90 ± 0.02	1.91 ± 0.05	1.90 ± 0.02

Note: CON, control group; HS, heat stress group; HS-MCE, Heat Stress plus MCE group; BW, body weight; ADFI, average daily feed intake; ADG, average daily gain; FCR, feed conversion ratio. Values are expressed as means ± SEM, *n* = 6. ^a–c^ Means within a row with different superscripts are different at *p* < 0.05.

**Table 3:** Effects of HS and MCE on organ indices of yellow-feathered broilers.

Item (% BW)	Treatment		
	CON	HS	HS-MCE
Bursa index	0.24 ± 0.03	0.18 ± 0.02	0.19 ± 0.02
Spleen index	0.19 ± 0.007 ^a^	0.14 ± 0.004 ^c^	0.16 ± 0.007 ^b^
Liver index	2.51 ± 0.06 ^a^	2.18 ± 0.10 ^b^	2.44 ± 0.11 ^a^

Note: CON, control group; HS, heat stress group; HS-MCE, Heat Stress plus MCE group. Values are expressed as means ± SEM, *n* = 6. ^a–c^ Means within a row with different superscripts are different at *p* < 0.05.

**Table 4:** Effects of HS and MCE on biochemical indices of yellow-feathered broilers.

Item	Treatment		
	CON	HS	HS-MCE
Uric acid (mmol/L)	184.50 ± 11.20	207.67 ± 16.59	208.33 ± 20.07
Creatinine (mmol/L)	3.14 ± 0.54 ^ab^	4.49 ± 0.38 ^a^	2.27 ± 0.61 ^b^
AST(U/L)	252.01 ± 10.31 ^b^	283.89 ± 6.43 ^a^	255.00 ± 9.85 ^b^
ALT(U/L)	7.40 ± 0.90 ^b^	11.55 ± 1.14 ^a^	8.05 ± 0.54 ^b^
ALP(U/L)	1657.56 ± 64.57 ^a^	1307.57 ± 144.14 ^b^	1880.54 ± 113.55 ^a^
LDH(U/L)	1191.33 ± 100.99 ^b^	1729.17 ± 108.84 ^a^	1297.33 ± 88.89 ^b^
TP (g/L)	35.89 ± 1.41	33.17 ± 1.30	36.71 ± 1.60
TC (mmol/L)	3.04 ± 0.26 ^b^	3.60 ± 0.15 ^a^	3.20 ± 0.09 ^ab^
LDL-C (mmol/L)	0.98 ± 0.07 ^b^	1.16 ± 0.04 ^a^	1.02 ± 0.06 ^ab^
HDL-C (mmol/L)	2.27 ± 0.12	1.98 ± 0.16	2.22 ± 0.05
GLU, mol/L	9.28 ± 0.24 ^a^	8.09 ± 0.37 ^b^	9.24 ± 0.45 ^a^

Note: CON, control group; HS, heat stress group; HS-MCE, Heat Stress plus MCE group; AST, Aspartate aminotransferase; ALT, Alanine aminotransferase; ALP, Alkaline phosphatase; LDH, Lactate dehydrogenase; TP, Total protein; TC, Cholesterol; LDL-C, Low density lipoprotein; HDL-C, High density lipoprotein; GLU, glucose. Values are expressed as means ± SEM, *n* = 6. ^a,b^ Means within a row with different superscripts are different at *p* < 0.05.

## Data Availability

The data presented in this study are available on request from the corresponding author.

## References

[1] Wang J., Xue X., Liu Q., Zhang S., Peng M., Zhou J., Chen L., Fang F. (2019). Effects of duration of thermal stress on growth performance, serum oxidative stress indices, the expression and localization of ABCG2 and mitochondria ROS production of skeletal muscle, small intestine and immune organs in broilers. J. Therm. Biol..

[2] Nawab A., Ibtisham F., Li G., Kieser B., Wu J., Liu W., Zhao Y., Nawab Y., Li K., Xiao M. (2018). Heat stress in poultry production: Mitigation strategies to overcome the future challenges facing the global poultry industry. J. Therm. Biol..

[3] Mohammed A., Jacobs J., Murugesan G., Cheng H. (2018). Effect of dietary synbiotic supplement on behavioral patterns and growth performance of broiler chickens reared under heat stress. Poult. Sci..

[4] Patra A.K., Kar I. (2021). Heat stress on microbiota composition, barrier integrity, and nutrient transport in gut, production performance, and its amelioration in farm animals. J. Anim. Sci. Technol..

[5] Song Z., Cheng K., Zheng X., Ahmad H., Zhang L., Wang T. (2018). Effects of dietary supplementation with enzymatically treated Artemisia annua on growth performance, intestinal morphology, digestive enzyme activities, immunity, and antioxidant capacity of heat-stressed broilers. Poult. Sci..

[6] Cheng K., Zhang M., Huang X., Zheng X., Song Z., Zhang L., Wang T. (2018). An evaluation of natural and synthetic vitamin E supplementation on growth performance and antioxidant capacity of broilers in early age. Can. J. Anim. Sci..

[7] Liu G., Zhu H., Ma T., Yan Z., Zhang Y., Geng Y., Zhu Y., Shi Y. (2020). Effect of chronic cyclic heat stress on the intestinal morphology, oxidative status and cecal bacterial communities in broilers. J. Therm. Biol..

[8] Bercik P., Collins S.M., Verdu E.F. (2012). Microbes and the gut-brain axis. Neurogastroenterol. Motil..

[9] Cao C., Chowdhury V.S., Cline M.A., Gilbert E.R. (2021). The Microbiota-Gut-Brain Axis During Heat Stress in Chickens: A Review. Front. Physiol..

[10] Liu W., Yuan Y., Sun C., Balasubramanian B., Zhao Z., An L. (2019). Effects of Dietary Betaine on Growth Performance, Digestive Function, Carcass Traits, and Meat Quality in Indigenous Yellow-Feathered Broilers under Long-Term Heat Stress. Animals.

[11] Liu W., Yuan Y., Sun C., Balasubramanian B., Zhao Z., An L. (2021). Effects of Dietary Supplementation of Algae-Derived Polysaccharides on Morphology, Tight Junctions, Antioxidant Capacity and Immune Response of Duodenum in Broilers under Heat Stress. Animals.

[12] Liu W.-C., Huang M.-Y., Balasubramanian B., Jha R. (2022). Heat Stress Affects Jejunal Immunity of Yellow-Feathered Broilers and Is Potentially Mediated by the Microbiome. Front. Physiol..

[13] Dong Z., Tang S.-S., Ma X.-L., Li C.-H., Tang Z.-S., Yang Z.-H., Zeng J.-G. (2022). Preclinical Safety Evaluation of Macleaya Cordata Extract: A Re-Assessment of General Toxicity and Genotoxicity Properties in Rodents. Front. Pharmacol..

[14] Dong Z., Liu M., Zhong X., Ou X., Yun X., Wang M., Ren S., Qing Z., Zeng J. (2021). Identification of the Impurities in Bopu Powder(^®^) and Sangrovit(^®^) by LC-MS Combined with a Screening Method. Molecules.

[15] Kumar G.S., Hazra S. (2014). Sanguinarine, a promising anticancer therapeutic: Photochemical and nucleic acid binding properties. RSC Adv..

[16] Hamoud R., Reichling J., Wink M. (2014). Synergistic antimicrobial activity of combinations of sanguinarine and EDTA with vancomycin against multidrug resistant bacteria. Drug Metab. Lett..

[17] Xue G.D., Wu S.B., Choct M., Pastor A., Steiner T., Swick R.A. (2017). Impact of a Macleaya cordata-derived alkaloid extract on necrotic enteritis in broilers. Poult. Sci..

[18] Wang P.-Q., Yin Z.-H., Kang W.-Y. (2013). Advance in studies on pharmacological activities of chelerythrine. Zhongguo Zhong Yao Za Zhi.

[19] Li W., Li H., Yao H., Mu Q., Zhao G., Li Y., Hu H., Niu X. (2014). Pharmacokinetic and anti-inflammatory effects of sanguinarine solid lipid nanoparticles. Inflammation.

[20] Hu N.-X., Chen M., Liu Y.-S., Shi Q., Yang B., Zhang H.-C., Cheng P., Tang Q., Liu Z.-Y., Zeng J.-G. (2019). Pharmacokinetics of sanguinarine, chelerythrine, and their metabolites in broiler chickens following oral and intravenous administration. J. Vet. Pharmacol. Ther..

[21] Liu Y.-L., Zhong L., Chen T., Shi Y., Hu Y., Zeng J.-G., Liu H.-Y., Xu S.-D. (2020). Dietary sanguinarine supplementation on the growth performance, immunity and intestinal health of grass carp (Ctenopharyngodon idellus) fed cottonseed and rapeseed meal diets. Aquaculture.

[22] Li Y., Li H., Chu Q., Xu F., Liang T., Zhou B. (2018). Macleaya cordata extracts suppressed the increase of a part of antibiotic resistance genes in fecal microorganism of weaned pigs. Can. J. Anim. Sci..

[23] Michels A., Neumann M., Leão G.F.M., Reck A.M., Bertagnon H.G., Lopes L.S., De Souza A.M., Dos Santos L.C., Júnior E.S.S. (2018). Isoquinoline alkaloids supplementation on performance and carcass traits of feedlot bulls. Asian-Australas J. Anim. Sci..

[24] Liu Z.-Y., Wang X.-L., Ou S.-Q., Hou D.-X., He J.-H. (2020). Sanguinarine modulate gut microbiome and intestinal morphology to enhance growth performance in broilers. PLoS ONE.

[25] Wang F., Yin Y., Yang M., Chen J., Fu C., Huang K. (2021). Effects of Combined Supplementation of Macleaya cordata Extract and Benzoic Acid on the Growth Performance, Immune Responses, Antioxidant Capacity, Intestinal Morphology, and Microbial Composition in Weaned Piglets. Front. Vet. Sci..

[26] Chen J., Kang B., Zhao Y., Yao K., Fu C. (2018). Effects of natural dietary supplementation with Macleaya cordata extract containing sanguinarine on growth performance and gut health of early-weaned piglets. J. Anim. Physiol. Anim. Nutr..

[27] He S., Li S., Arowolo M.A., Yu Q., Chen F., Hu R., He J. (2019). Effect of resveratrol on growth performance, rectal temperature and serum parameters of yellow-feather broilers under heat stress. Anim. Sci. J..

[28] Khadem A., Soler L., Everaert N., Niewold T.A. (2014). Growth promotion in broilers by both oxytetracycline and Macleaya cordata extract is based on their anti-inflammatory properties. Br. J. Nutr..

[29] Saeed M., Abbas G., Alagawany M., Kamboh A.A., Abd El-Hack M.E., Khafaga A.F., Chao S. (2019). Heat stress management in poultry farms: A comprehensive overview. J. Therm. Biol..

[30] Moustafa E., Alsanie W., Gaber A., Kamel N., Alaqil A., Abbas A. (2021). Blue-Green Algae (Spirulina platensis) Alleviates the Negative Impact of Heat Stress on Broiler Production Performance and Redox Status. Animals.

[31] Quinteiro-Filho W.M., Rodrigues M.V., Ribeiro A., Ferraz-De-Paula V., Pinheiro M.L., Sá L.R.M., Ferreira A.J.P., Palermo-Neto J. (2012). Acute heat stress impairs performance parameters and induces mild intestinal enteritis in broiler chickens: Role of acute hypothalamic-pituitary-adrenal axis activation. J. Anim. Sci..

[32] Mack L.A., Felver-Gant J.N., Dennis R.L., Cheng H.W. (2013). Genetic variations alter production and behavioral responses following heat stress in 2 strains of laying hens. Poult. Sci..

[33] He X., Lu Z., Ma B., Zhang L., Li J., Jiang Y., Zhou G., Gao F. (2018). Effects of chronic heat exposure on growth performance, intestinal epithelial histology, appetite-related hormones and genes expression in broilers. J. Sci. Food Agric..

[34] Guo S., Lei J., Liu L., Qu X., Li P., Liu X., Guo Y., Gao Q., Lan F., Xiao B. (2021). Effects of Macleaya cordata extract on laying performance, egg quality, and serum indices in Xuefeng black-bone chicken. Poult. Sci..

[35] Bussabong P., Rairat T., Chuchird N., Keetanon A., Phansawat P., Cherdkeattipol K., Pichitkul P., Kraitavin W. (2021). Effects of isoquinoline alkaloids from Macleaya cordata on growth performance, survival, immune response, and resistance to Vibrio parahaemolyticus infection of Pacific white shrimp (Litopenaeus vannamei). PLoS ONE.

[36] Chen K., Liu Y., Cheng Y., Yan Q., Zhou C., He Z., Zeng J., He J., Tan Z. (2020). Supplementation of Lactobacillus plantarum or Macleaya cordata Extract Alleviates Oxidative Damage Induced by Weaning in the Lower Gut of Young Goats. Animals.

[37] Yang Q., Wang Z., Cui Y., Sun R.-Y., Liang W.-W., Wang L.-J., Wang W.-Y., Lv Q., Hu J. (2019). Effects of Taurine on Bowel Inflammatory Factor of Small Intestinal Mucosa Impaired by Heat Stress in Broilers. Adv. Exp. Med. Biol..

[38] Gu J., Zhao L., Chen Y.-Z., Guo Y.-X., Sun Y., Guo Q., Duan G.-X., Li C., Tang Z.-B., Zhang Z.-X. (2022). Preventive effect of sanguinarine on intestinal injury in mice exposed to whole abdominal irradiation. Biomed Pharm..

[39] Niu X.-F., Zhou P., Li W.-F., Xu H.-B. (2011). Effects of chelerythrine, a specific inhibitor of cyclooxygenase-2, on acute inflammation in mice. Fitoterapia.

[40] Bitterman S., Ben Shahar Y., Pollak Y., Bitterman N., Halabi S., Coran A.G., Bitterman A., Sukhotnik I. (2017). Effect of Chelerythrine on Intestinal Cell Turnover following Intestinal Ischemia-Reperfusion Injury in a Rat Model. Eur. J. Pediatr. Surg..

[41] Gogoi S., Kolluri G., Tyagi J.S., Marappan G., Manickam K., Narayan R. (2021). Impact of heat stress on broilers with varying body weights: Elucidating their interactive role through physiological signatures. J. Therm. Biol..

[42] Guan G., Ding S., Yin Y., Duraipandiyan V., Al-Dhabi N.A., Liu G. (2019). Macleaya cordata extract alleviated oxidative stress and altered innate immune response in mice challenged with enterotoxigenic Escherichia coli. Sci. China Life Sci..

[43] He S., Yu Q., He Y., Hu R., Xia S., He J. (2019). Dietary resveratrol supplementation inhibits heat stress-induced high-activated innate immunity and inflammatory response in spleen of yellow-feather broilers. Poult. Sci..

[44] Chen Y., Cheng Y., Wen C., Zhou Y. (2020). Protective effects of dietary mannan oligosaccharide on heat stress-induced hepatic damage in broilers. Environ. Sci. Pollut. Res. Int..

[45] Bavarsadi M., Mahdavi A.H., Mahyari S.A., Jahanian E. (2017). Effects of different levels of sanguinarine on antioxidant indices, immunological responses, ileal microbial counts and jejunal morphology of laying hens fed diets with different levels of crude protein. J. Anim. Physiol. Anim. Nutr..

[46] Meng Y.-Y., Liu Y., Hu Z.-F., Zhang Y., Ni J., Ma Z.-G., Liao H.-H., Wu Q.-Q., Tang Q.-Z. (2018). Sanguinarine Attenuates Lipopolysaccharide-induced Inflammation and Apoptosis by Inhibiting the TLR4/NF-κB Pathway in H9c2 Cardiomyocytes. Curr. Med. Sci..

[47] Elazab S., Elshater N., Kishaway A., Ei-Emam H. (2021). Cinnamon Extract and Probiotic Supplementation Alleviate Copper-Induced Nephrotoxicity via Modulating Oxidative Stress, Inflammation, and Apoptosis in Broiler Chickens. Animals.

[48] Zhou F., Yu G., Wang G., Liu Y., Zhang L., Wang W., Chen N. (2019). Association of serum uric acid levels with the incident of kidney disease and rapid eGFR decline in Chinese individuals with eGFR > 60 mL/min/1.73 m(2) and negative proteinuria. Clin. Exp. Nephrol..

[49] Tang S., Zhou S., Yin B., Xu J., Di L., Zhang J., Bao E. (2018). Heat stress-induced renal damage in poultry and the protective effects of HSP60 and HSP47. Cell Stress Chaperones.

[50] Lamp O., Derno M., Otten W., Mielenz M., Nürnberg G., Kuhla B. (2015). Metabolic Heat Stress Adaption in Transition Cows: Differences in Macronutrient Oxidation between Late-Gestating and Early-Lactating German Holstein Dairy Cows. PLoS ONE.

[51] Liu G., Aguilar Y.M., Zhang L., Ren W., Chen S., Guan G., Xiong X., Liao P., Li T., Huang R. (2016). Dietary supplementation with sanguinarine enhances serum metabolites and antibodies in growing pigs. J. Anim. Sci..

[52] Dršata J., Ulrichová J., Walterová D. (1996). Sanguinarine and Chelerythrine as Inhibitors of Aromatic Amino Acid Decarboxylase. J. Enzym. Inhib..

[53] Mellor S. (2001). Natural appetisers from plants. Feed. Mix..

[54] Vieira S.L., Oyarzabal O., Freitas D.M., Berres J., Peña J.E.M., Torres C.A., Coneglian J.L.B. (2008). Performance of Broilers Fed Diets Supplemented with Sanguinarine-Like Alkaloids and Organic Acids1. J. Appl. Poult. Res..

[55] Herbert J.-M., Savi P., Laplace M.-C., Dumas A., Dol F. (1993). Chelerythrine, a selective protein kinase C inhibitor, counteracts pyrogen-induced expression of tissue factor without effect on thrombomodulin down-regulation in endothelial cells. Thromb. Res..

[56] Nowak G., Takacsova-Bakajsova D., Megyesi J. (2017). Deletion of protein kinase C-ε attenuates mitochondrial dysfunction and ameliorates ischemic renal injury. Am. J. Physiol. Renal. Physiol..

[57] Ozer E.K., Goktas M.T., Kilinc I., Bariskaner H., Ugurluoglu C., Iskit A.B. (2017). Celecoxib administration reduced mortality, mesenteric hypoperfusion, aortic dysfunction and multiple organ injury in septic rats. Biomed Pharm..

[58] Hosseini-Vashan S.J., Golian A., Yaghobfar A. (2016). Growth, immune, antioxidant, and bone responses of heat stress-exposed broilers fed diets supplemented with tomato pomace. Int. J. Biometeorol..

[59] Wan X., Jiang L., Zhong H., Lu Y., Zhang L., Wang T. (2017). Effects of enzymatically treated Artemisia annua L. on growth performance and some blood parameters of broilers exposed to heat stress. Anim. Sci. J..

[60] Ke W., Lin X., Yu Z., Sun Q., Zhang Q. (2017). Molluscicidal activity and physiological toxicity of Macleaya cordata alkaloids components on snail Oncomelania hupensis. Pestic. Biochem. Physiol..

[61] Liu X.-W., Tang C.-L., Zheng H., Wu J.-X., Wu F., Mo Y.-Y., Liu X., Zhu H.-J., Yin C.-L., Cheng B. (2018). Investigation of the hepatoprotective effect of Corydalis saxicola Bunting on carbon tetrachloride-induced liver fibrosis in rats by (1)H-NMR-based metabonomics and network pharmacology approaches. J. Pharm. Biomed. Anal..

[62] Huang C.-Y., Huang Y.-J., Zhang Z.-Y., Liu Y.-S., Liu Z.-Y. (2021). Metabolism and Tissue Distribution of Chelerythrine and Effects of Macleaya Cordata Extracts on Liver NAD(P)H Quinone Oxidoreductase. Front. Vet. Sci..

[63] Zeng J., Xiao L., Wang Y., Liu L., Zhong M., He X., Liu Y. (2012). Experimental study on antagonizing liver fibrosis of Macleaya cordata extract. Chin. J. Exp. Tradit. Med. Formulae.

[64] Luo J., Song J., Liu L., Xue B., Tian G., Yang Y. (2018). Effect of epigallocatechin gallate on growth performance and serum biochemical metabolites in heat-stressed broilers. Poult. Sci..

[65] Ryu S.-T., Park B.-S., Bang H.-T., Kang H.-K., Hwangbo J. (2016). Effects of anti-heat diet and inverse lighting on growth performance, immune organ, microorganism and short chain fatty acids of broiler chickens under heat stress. J. Environ. Biol..

[66] Bagath M., Krishnan G., Devaraj C., Rashamol V., Pragna P., Lees A., Sejian V. (2019). The impact of heat stress on the immune system in dairy cattle: A review. Res. Vet. Sci..

[67] Kuo T., Harris C.A., Wang J.-C. (2013). Metabolic functions of glucocorticoid receptor in skeletal muscle. Mol. Cell. Endocrinol..

[68] Xie J., Tang L., Lu L., Zhang L., Lin X., Liu H.-C., Odle J., Luo X. (2015). Effects of acute and chronic heat stress on plasma metabolites, hormones and oxidant status in restrictedly fed broiler breeders. Poult. Sci..

[69] Dvořák Z., Vrzal R., Maurel P., Ulrichová J. (2006). Differential effects of selected natural compounds with anti-inflammatory activity on the glucocorticoid receptor and NF-kappaB in HeLa cells. Chem. Biol. Interact..

[70] Shang Y., Kumar S., Oakley B., Kim W.K. (2018). Chicken Gut Microbiota: Importance and Detection Technology. Front. Vet. Sci..

[71] Gong L., Xiao G., Zheng L., Yan X., Qi Q., Zhu C., Feng X., Huang W., Zhang H. (2021). Effects of Dietary Tributyrin on Growth Performance, Biochemical Indices, and Intestinal Microbiota of Yellow-Feathered Broilers. Animals.

[72] Wang X., Feng J., Zhang M., Li X., Ma D., Chang S. (2018). Effects of high ambient temperature on the community structure and composition of ileal microbiome of broilers. Poult. Sci..

[73] Shi D., Bai L., Qu Q., Zhou S., Yang M., Guo S., Li Q., Liu C. (2019). Impact of gut microbiota structure in heat-stressed broilers. Poult. Sci..

[74] Zhu L., Liao R., Wu N., Zhu G., Yang C. (2019). Heat stress mediates changes in fecal microbiome and functional pathways of laying hens. Appl. Microbiol. Biotechnol..

[75] Rios-Covian D., Salazar N., Gueimonde M., de los Reyes-Gavilan C.G. (2017). Shaping the Metabolism of Intestinal Bacteroides Population through Diet to Improve Human Health. Front. Microbiol..

[76] Huang Y., Lv H., Song Y., Sun C., Zhang Z., Chen S. (2021). Community composition of cecal microbiota in commercial yellow broilers with high and low feed efficiencies. Poult. Sci..

[77] Torok V.A., Hughes R.J., Mikkelsen L.L., Perez-Maldonado R., Balding K., MacAlpine R., Percy N.J., Ophel-Keller K. (2011). Identification and characterization of potential performance-related gut microbiotas in broiler chickens across various feeding trials. Appl. Environ. Microbiol..

[78] Tavella T., Rampelli S., Guidarelli G., Bazzocchi A., Gasperini C., Pujos-Guillot E., Comte B., Barone M., Biagi E., Candela M. (2021). Elevated gut microbiome abundance of Christensenellaceae, Porphyromonadaceae and Rikenellaceae is associated with reduced visceral adipose tissue and healthier metabolic profile in Italian elderly. Gut Microbes.

[79] Hart M.L., Ericsson A.C., Franklin C.L. (2017). Differing Complex Microbiota Alter Disease Severity of the IL-10(-/-) Mouse Model of Inflammatory Bowel Disease. Front. Microbiol..

[80] Latorre J.D., Adhikari B., Park S.H., Teague K.D., Graham L.E., Mahaffey B.D., Baxter M.F.A., Hernandez-Velasco X., Kwon Y.M., Ricke S.C. (2018). Evaluation of the Epithelial Barrier Function and Ileal Microbiome in an Established Necrotic Enteritis Challenge Model in Broiler Chickens. Front. Vet. Sci..

[81] Biddle A., Stewart L., Blanchard J.L., Leschine S. (2013). Untangling the Genetic Basis of Fibrolytic Specialization by Lachnospiraceae and Ruminococcaceae in Diverse Gut Communities. Microb. Ecol. Divers..

[82] Van Immerseel F. (2003). Invasion of Salmonella enteritidis in avian intestinal epithelial cells in vitro is influenced by short-chain fatty acids. Int. J. Food Microbiol..

[83] Rostagno M.H. (2020). Effects of heat stress on the gut health of poultry. J. Anim. Sci..

[84] Zhang J.F., Hu Z.P., Lu C.H., Yang M.X., Zhang L.L., Wang T. (2015). Dietary curcumin supplementation protects against heat-stress-impaired growth performance of broilers possibly through a mitochondrial pathway. J. Anim. Sci..

[85] Farzi A., Fröhlich E.E., Holzer P. (2018). Gut Microbiota and the Neuroendocrine System. Neurotherapeutics.

[86] Le Sciellour M., Zemb O., Hochu I., Riquet J., Gilbert H., Giorgi M., Billon Y., Gourdine J.-L., Renaudeau D. (2019). Effect of chronic and acute heat challenges on fecal microbiota composition, production, and thermoregulation traits in growing pigs1,2. J. Anim. Sci..

[87] Sohail M.U., Hume M.E., Byrd J.A., Nisbet D.J., Shabbir M.Z., Ijaz A., Rehman H. (2015). Molecular analysis of the caecal and tracheal microbiome of heat-stressed broilers supplemented with prebiotic and probiotic. Avian. Pathol..

[88] Xing S., Wang X., Diao H., Zhang M., Zhou Y., Feng J. (2019). Changes in the cecal microbiota of laying hens during heat stress is mainly associated with reduced feed intake. Poult. Sci..

[89] Goel A., Kim B.-J., Ncho C.-M., Jeong C.-M., Gupta V., Jung J.-Y., Ha S.-Y., Lee D.-H., Yang J.-K., Choi Y.-H. (2021). Dietary Supplementation of Shredded, Steam-Exploded Pine Particles Decreases Pathogenic Microbes in the Cecum of Acute Heat-Stressed Broilers. Animals.

[90] Zhang S., Wu P., Wang Q., Ling H., Jiang S., Zhang G. (2018). Effects of veterinary boluohui powder on the growth of commonly used probiotics. Feed. Ind..

